# MXene-Integrated Microneedle Patches with Innate Molecule Encapsulation for Wound Healing

**DOI:** 10.34133/2021/9838490

**Published:** 2021-06-30

**Authors:** Lingyu Sun, Lu Fan, Feika Bian, Guopu Chen, Yuetong Wang, Yuanjin Zhao

**Affiliations:** ^1^Department of Rheumatology and Immunology, The Affiliated Drum Tower Hospital of Nanjing University Medical School, 210008 Nanjing, China; ^2^State Key Laboratory of Bioelectronics, School of Biological Science and Medical Engineering, Southeast University, Nanjing 210096, China

## Abstract

Wound healing is a complex physiological process that involves coordinated phases such as inflammation and neovascularization. Attempts to promote the healing process tend to construct an effective delivery system based on different drugs and materials. In this paper, we propose novel MXene-integrated microneedle patches with adenosine encapsulation for wound healing. Owing to the dynamic covalent bonding capacity of boronate molecules with adenosine, 3-(acrylamido)phenylboronic acid- (PBA-) integrated polyethylene glycol diacrylate (PEGDA) hydrogel is utilized as the host material of microneedle patches. Benefitting from photothermal conversion capacity of MXene, the release of loaded adenosine could be accelerated under NIR irradiation for maintaining the activation signal around injury site. In vitro cell experiments proved the effect of MXene-integrated microneedle patches with adenosine encapsulation in enhancing angiogenesis. When applied for treating animal models, it is demonstrated that the microneedle patches efficiently promote angiogenesis, which is conductive to wound healing. These features make the proposed microneedle patch potential for finding applications in wound healing and other biomedical fields.

## 1. Introduction

Wound treatment, which involves complex repair processes, has become a healthcare concern and economic burden around the world [1, 2]. Given that, various therapies have been developed to promote wound healing, such as pharmacologic intervention, cell transplantation, and artificial substitutes [3–9]. Among them, pharmacologic intervention using chemical or biological agents is one of the most popular methods, owing to its effectiveness and low cost. In this therapy, diverse drug delivery systems based on biomaterials with different morphology designs including nano- or microparticles, microfibers, and patches have been proposed to prolong release time and decrease potential toxicities of drugs [10–16]. Microneedles, which could penetrate the skin in a noninvasive and painless manner, are receiving great interest and research in constructing drug delivery systems [17–21]. Up to date, microneedle-based drug delivery systems have shown effect on treating multifarious diseases like diabetes, osteoarthritis, and wound healing [22–27]. Despite many successes, most of the microneedles are with simple release strategies and have difficulty in controllable active delivery. In addition, the common drugs utilized in these systems are synthetic compounds, while the employment of natural molecules for wound healing has been rarely explored. Therefore, it is still desirable to develop new microneedles with biosynthetic active encapsulations for efficient wound healing.

**Figure 1:**
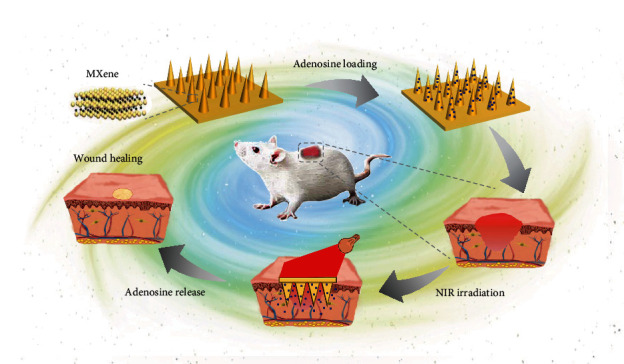
Schematic illustration. Schematic diagram illustrating the mechanism of MXene-integrated microneedle patches with adenosine encapsulation for wound healing.

Herein, we presented a novel MXene-integrated microneedle patch with adenosine encapsulation for wound healing, as schemed in Figure 1. Adenosine is recognized as an active ingredient ubiquitously in the body, which could regulate cellular behavior by activating G-protein-coupled adenosine receptors [28]. Once the tissue is injured, the local concentration of adenosine would increase to accelerate the repair process. Recent studies have demonstrated the value of adenosine in neovascularization and fibrosis, which indicates that adenosine is an ideal candidate for wound healing [28, 29]. In comparison, MXene, referring to transition metal carbides and nitride, is an emerging two-dimensional material family that exhibits tremendous potential in various fields, including energy storage [30, 31], catalysis [32, 33], biomedical applications [34–36], etc. [37]. In particular, MXene has aroused attention in constructing intelligent drug delivery systems for its prominent photothermal conversion capacity [38, 39]. However, MXene-based delivery microneedles have seldom been explored. In addition, effective integration of adenosine into biomaterials with controllable release capability is still a challenge.

In this paper, we developed the desired microneedle patches by using a boronate- and MXene-mixed hydrogel to replicate a microwell template. As boronate molecules possess the capacity of dynamically covalent bonding with adenosine, 3-(acrylamido)phenylboronic acid- (PBA-) integrated polyethylene glycol diacrylate (PEGDA) hydrogel was employed as the host material to construct the patches. The derived PBA patches not only could deliver exogenous adenosine but also had the capacity of sequestering endogenous adenosine for injury repair. Upon near infrared (NIR) irradiation, MXene could rapidly convert light into thermal energy and thus accelerate the release of loaded adenosine for maintaining the activation signal around the injury site. In vitro cell experiments revealed the effect of MXene-integrated microneedle patches with adenosine encapsulation in enhancing angiogenesis. When applied for animal models, it was demonstrated that the functional microneedle patches efficiently helped in promoting angiogenesis, thus accelerating the wound healing process. Thus, we conceive that the innate molecule-encapsulated microneedle patch with photothermal response would open a chapter of exploiting effective therapies for wound healing and other diseases.

## 2. Results

**Figure 2:**
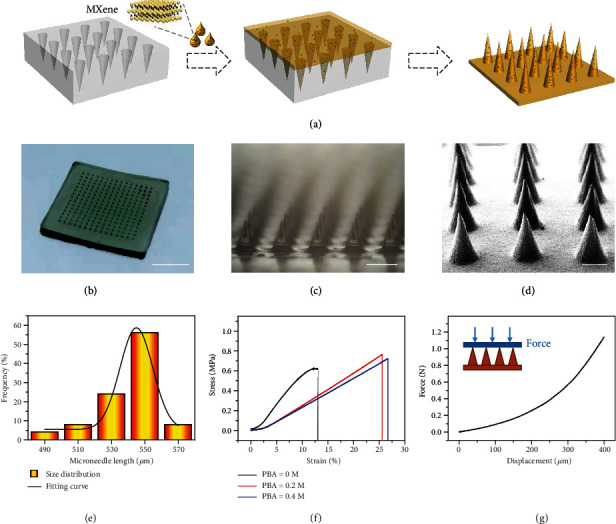
Fabrication and characterization of hybrid microneedle patch. (a) Schematic diagram showing the fabrication of microneedle patches. (b) Optical image of the MXene-integrated hydrogel microneedle patch. (c) Microscopy image magnifying the morphology of microneedle array. (d) Scanning electron microscope (SEM) image of the microneedle patch. (e) Uniformity statistics of the microneedle array. (f) Stress-strain curves of the hydrogel patch mixed with different concentrations of PBA. (g) Compressive force test of the microneedle patch. Scale bars are 4 mm in (b), 400 *μ*m in (c), and 200 *μ*m in (d).

In a typical experiment, the desired microneedle patches were fabricated from a template replication strategy, as shown in Figure 2(a). The 3-(acrylamido)phenylboronic acid- (PBA-) doped polyethylene glycol diacrylate (PEGDA) hydrogel precursor solution containing MXene was added on a polydimethylsiloxane- (PDMS-) negative multiwell mold, followed by a centrifugation treatment procedure to make sure that the solution filled up the voids. After ultraviolet (UV) irradiation and template removal, the MXene-integrated PBA hydrogel patch with cone-shaped microneedle array was successfully achieved (Figures 2(b) and 2(c)), while the field emission scanning electron microscope (FESEM) images presented consistent results (Figure 2(d)). The derived microneedle array showed uniform size distribution, which indicated the feasibility of the template method for fabrication of microneedle patches, as shown in Figure 2(e). Besides, the elemental analysis was conducted, which proved the successful integration of PBA and MXene into the hydrogel (Figure S1a, S1b). Also, according to Raman spectrum results, the characteristic peaks of the benzene ring were observed at 1000 cm^−1^ and near 1600 cm^−1^ (Figure S1c). In addition, the mechanical intensity test of the PBA hybrid hydrogel was implemented. The results displayed that with the increase of PBA concentration, the stretchability of the hydrogel was improved (Figure 2(f)), while the increase of the PEGDA concentration would enhance the mechanical intensity of the composite hydrogel (Figure S1d). Meanwhile, the microneedle patch could withstand compressive forces, as shown in Figure 2(g).

**Figure 3:**
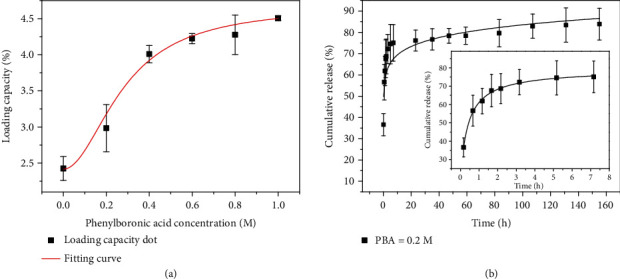
Sequestration and release capacity evaluation. (a) Adenosine loading capacity of hydrogels with different PBA concentrations. (b) Cumulative release curves of adenosine from the hybrid hydrogel for days (insert is the cumulative release curve during the first 7 hours).

To investigate the performance of PBA-integrated hydrogel for sequestering and releasing adenosine, a series of PEGDA hydrogels with different amounts of PBA were prepared. With the assistance of the UV/vis technique, a standard curve of adenosine concentration was measured, as shown in Figure S2. Within the range from 0.001 mg/mL to 0.09 mg/mL, the absorbance value showed a linear relationship with adenosine concentration. To examine the loading capacity of adenosine, PBA-integrated hydrogels were incubated in solutions with excess adenosine overnight. After thorough cleaning, the bound adenosine was released from the PBA/PEGDA hydrogel to the acetic acid solution for quantification. With the increase of PBA concentration, the adenosine loading capacity increased correspondingly (Figure 3(a)), which may be attributed to the dynamic covalent bonding capacity of boronate molecules with adenosine. The stability of the resultant borate esters is affected by various factors, such as pH value and solution composition [40]. When the environmental concentration was low, the bound adenosine would be released from the hydrogel to reach equilibrium. It was demonstrated that the adenosine underwent a rapid release period during the first hours and then slowed down for the following days (Figure 3(b)). These features demonstrated the potential value of the PBA-integrated hydrogel patch for wound healing.

**Figure 4:**
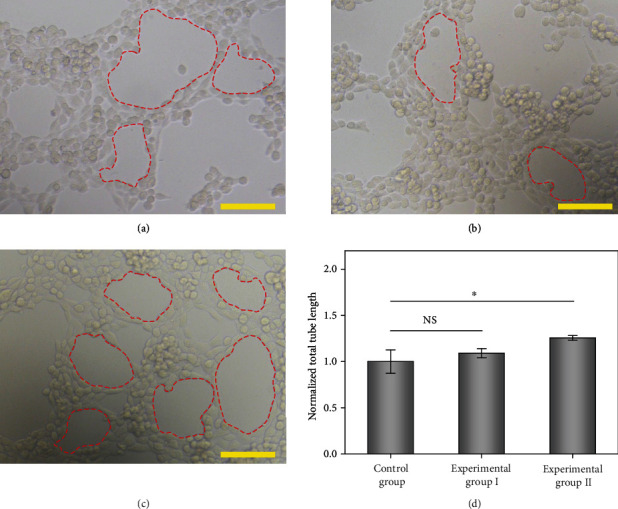
Angiogenic effect evaluation results. (a–c) Optical images showing the HUVECs cultured in different groups: (a) control group, (b) experimental group I, and (c) experimental group II. The red dotted areas represent formed tubular structures. Scale bars are 100 *μ*m. (d) Statistic graph of the total tube length of three groups (*n* = 3). Error bars represent standard deviation. 0.01 <  ^∗^*p* < 0.05, NS: not significant.

MTT assay was then performed to verify the biocompatibility of the adenosine-encapsulated microneedle patch. The results showed that the MXene-integrated PBA hydrogel had no negative effect on the growth of cells, as shown in Figure S3. Adenosine, an active and common ingredient in organisms, has demonstrated value in promoting the formation of blood vessels. To evaluate the angiogenic action of adenosine in vitro, human umbilical vein ECs (HUVECs) were utilized as model cells. In this research, HUVECs were divided into three groups, that is, the control group (cultured in a well plate), experimental group I (cultured in a well plate with an unloaded hydrogel), and experimental group II (cultured in a well plate with an adenosine-loaded hydrogel). After 4 hours of cultivation, tubular structures in these groups were observed and recorded, as shown in Figure 4. During the process, NIR irradiation was carried out to accelerate the release of adenosine. According to the statistical results, the total tube length in experimental group II was higher than that in the other two groups, attributed to the angiogenic effect of adenosine. These results preliminarily proved the potential of our adenosine-encapsulated microneedle patch for wound healing.

**Figure 5:**
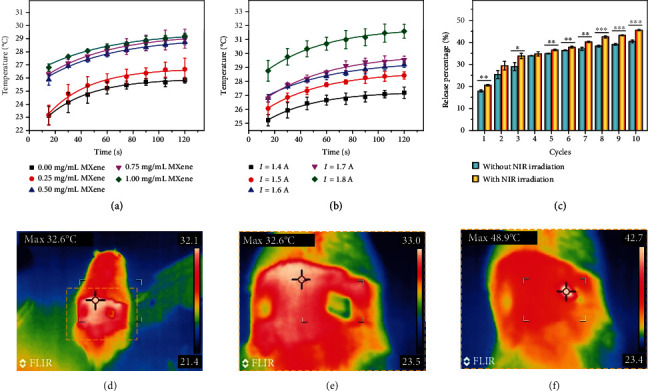
Photothermal test of MXene-integrated hydrogel and its release profile. (a) Temperature-time curves of the microneedle patch under NIR irradiation with different MXene concentrations (in PBS solution). (b) Temperature-time curves of the microneedle patch under different NIR irradiation intensities (in PBS solution). (c) The cumulative release of adenosine from PBA-integrated hydrogel with/without 10 cycles of NIR irradiation. ^∗^*p* < 0.05, ^∗∗^*p* < 0.01, and ^∗∗∗^*p* < 0.001. (d–f) Thermal images of the MN patch on rat skin (d, e) before and (f) after NIR irradiation.

Because of the excellent photothermal transition capacity of MXene, the composite hydrogel could convert light into thermal energy upon NIR irradiation. To demonstrate this, photothermal capacity of the MXene-integrated microneedle patch in a liquid environment was first investigated, as shown in Figures 5(a) and 5(b). With the increase of rradiation time, the temperature of the patch gradually increased until plateau. During the process, the heating up speed showed a positive relationship with MXene concentration and NIR irradiation intensity. Based on this feature, cumulative release profiles of adenosine with and without programmed NIR irradiation were investigated. Compared with the group without NIR irradiation, the release of adenosine from the MXene-integrated microneedle patch could be accelerated upon NIR irradiation (Figure 5(c)). Data analysis revealed that there was significant difference between these two groups after several cycles of NIR irradiation. After 10 cycles, the release percentage of irradiation group reached 45.5%, while another group was about 40%. When the MXene-integrated microneedle patch was placed in the air, the photothermal conversion efficiency was enhanced (Figure S4). Similarly, when the composite patch was applied to the dorsal skin of rats, the local temperature around the patch could rapidly rise from about 30°C to about 50°C (Figures 5(d)–5(f)). These results proved the photoresponsive capacity of the MXene-integrated microneedle patch, which made it the ideal choice to construct an intelligent drug delivery system for a standby injury repair experiment.

**Figure 6:**
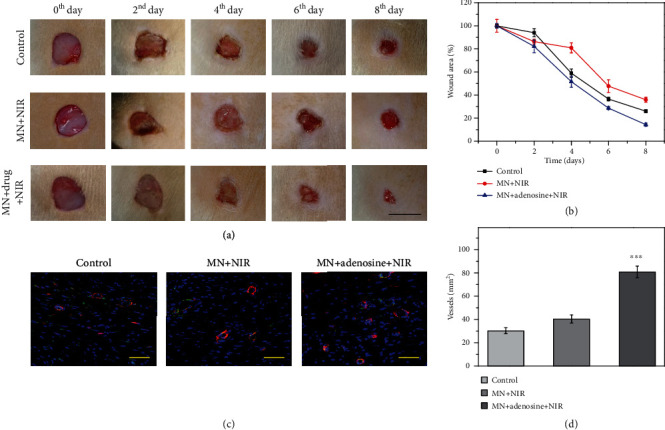
Wound healing characterization. (a) Representative images showing the wound healing process in different groups: control group, MN+NIR group, and MN+adenosine+NIR group, respectively. (b) Quantitative analysis of wound area in recovery process. (c) Immunofluorescence staining showing the neovascularization situation. CD31+ structures (green) were surrounded by *α*-smooth muscle actin-positive cells (red), indicating the formation of vascular ducts. (d) Quantification of vascular structures. The scale bars are 1 cm in (a) and 50 *μ*m in (c). ^∗∗∗^*p* < 0.001 when compared with the control group and MN+NIR group.

To evaluate the practical value of an adenosine-loaded microneedle patch in vivo, animal experiments were conducted by artificially producing wounds on rats. The rats were randomly divided into five groups that were treated with a phosphate buffer solution (control group), PBA/PEGDA microneedle patch without adenosine loading (MN group), PBA/PEGDA microneedle patch encapsulated with adenosine (MN+adenosine group), empty PBA/PEGDA microneedle patch with regular NIR irradiation (MN+NIR group), and adenosine-loaded PBA/PEGDA microneedle patch under regular NIR irradiation (MN+adenosine+NIR group). Because of their specific microstructure, microneedles could ensure adherence to wound sites through increasing the roughness of the patch. In addition, the existence of microneedles further enhanced the surface area of hydrogels for wound treatment. The wound recovery processes of these groups were recorded, and the wound areas were calculated at day 0, day 2, day 4, day 6, and day 8. It was demonstrated that MN+adenosine groups performed better in accelerating wound healing than MN groups and the control group, as shown in Figures 6(a) and 6(b) and Figure S5. In addition, the MN+adenosine+NIR group showed a more superior curative effect than the MN+adenosine group, which could be attributed to the promoted adenosine release under NIR irradiation.

During tissue repair process, angiogenesis was also an important indicator to reflect the function recovery condition. Herein, double immunofluorescence staining of CD31 and *α*-smooth muscle actin (*α*-SMA) was conducted to reflect the angiogenesis degree at wound sites, as shown in Figure 6(c) and Figure S6. CD31 and *α*-SMA are two typical markers of the vascular endothelial cell and the vascular smooth muscle cell, respectively. Representative fluorescent images revealed that the MN+adenosine+NIR group showed more intensive vascular structures at the wound site compared to the other groups, which embodied the positive role of adenosine in promoting angiogenesis. Moreover, the quantitative vascular analysis also showed the same tendency, as shown in Figure 6(d). These results, together with the above-mentioned features, demonstrated the practical value of the adenosine-integrated microneedle patch in promoting wound healing.

## 3. Discussion

In summary, we have presented a novel drug delivery system based on MXene-integrated microneedle patches encapsulated with adenosine. Based on the negative templates, microneedles with controllable morphology were successfully fabricated. Because of the existence of PBA molecules, adenosine could be effectively loaded in the hydrogel network. In vitro experiments revealed that the adenosine-contained microneedle patch achieved a sustained release, which was conducive to maintaining the activation signal around the injury site. Because of the integration of MXene, the regional temperature would increase and thus accelerate the release process of adenosine upon NIR irradiation for better treatment effect. It was worth mentioning that the released adenosine has a positive effect on promoting the formation of tubular structures. When applied for animal models, it has been demonstrated that the adenosine-encapsulated microneedle patch efficiently helped in angiogenesis and wound healing. According to previous studies, adenosine and its receptors could facilitate the wound repair process by promoting fibrosis, matrix production, and angiogenesis. Up to date, most of the existing microneedles depend on passive diffusion of synthetic drugs for treatment, which greatly limited their practical values in the clinic. In contrast, our proposed system showed superiority in biosynthetic active encapsulations and controllable release. However, different diseases have different requirements on the functions of biomedical patches, such as drug delivery, hemostasis, and biodegradability. Based on these demands, microneedle patches with a more delicate structure and abundant functionalities would be developed and open a chapter for exploiting effective therapies of wound healing and other diseases.

## 4. Materials and Methods

### 4.1. Materials

3-(Acrylamido)phenylboronic acid (PBA, 98%), adenosine (suitable for cell culture), photoinitiator 2-hydroxy-2-methylpropiophenone (HMPP, 97%), and polyethylene glycol diacrylate (PEGDA, average Mn 700) were from Sigma-Aldrich. MXene (Ti_3_C_2_, 5 mg/mL) was obtained from the Nanjing XF Nano Material Tech Co., Ltd. (Nanjing, China). Ethanol (AR) was bought from the China National Pharmaceutical Group Corporation. Deionized water with a resistivity of 18.2 M*Ω*·cm was utilized in all experiments. All chemical reagents were of the available analytical grade and used as received.

### 4.2. Fabrication of the Composite Microneedle Patch

20% (*v*/*v*) PEGDA, 30% (*v*/*v*) ethanol, 1% (*v*/*v*) HMPP, MXene (0 mg/mL-1 mg/mL), and PBA (0 M-1 M) were thoroughly mixed in deionized water. Subsequently, the mixed solution was added in a negative multiwell template and centrifuged at 2000 rpm to fill the cavities. After removing the redundant solution, the microneedle patch could be achieved after exposing the solution-filled template to UV (365 nm) irradiation for about 10 s. Finally, the patch was peeled off and cleaned by the mixture of water and ethanol for the following experiments.

### 4.3. Mechanical Strength Test

To achieve the stress-strain curves, the both ends of patches were fixed and stretched at a speed of 10 mm/min. For the compressive force test, the microneedle patch was placed on a displacement-force test system with microneedle tips facing upward. After that, the force sensor then approached the microneedle patch at the speed of 1 mm/min. When the sensor was in contact with the microneedle tips, force measurement began and ended at the distance of 0.4 mm.

### 4.4. In Vitro Adenosine Loading

The MXene-integrated PBA patches were soaked in saturated adenosine solution (10 mg/mL) for in vitro loading, and unbounded adenosine was washed thoroughly for the following measurement. To verify the loading efficiency, the adenosine-loaded hydrogel was soaked in an acetate buffer (0.1 M, pH 4.5) for 2 h to release adenosine in the buffer and then measured by UV/vis at 260 nm. The corresponding concentrations were obtained by referring to a standard curve that measured from a series of adenosine solutions with known concentrations (0.01 mg/mL-0.09 mg/mL).

### 4.5. In Vitro Adenosine Release

To investigate the adenosine release capacity, the PBA patches with adenosine loading were incubated and shook in 2 mL PBS solution (37°C, 300 rpm). At set intervals, a certain amount of the release medium (100 *μ*L) was removed from the container for UV/vis measurement, and the medium was replenished by 100 *μ*L fresh PBS solution.

### 4.6. Biocompatibility Test

NIH 3T3 cells were divided into three groups: a control group that was cultured in a blank 48-well plate, experimental group I cultured in a plate with MXene/PBA hydrogel, and experimental group II cultured in a plate with adenosine-loaded MXene/PBA hydrogel. For the daily test, 10% (*v*/*v*) MTT solution was added in the culture medium of different groups. After 4 h of cultivation and thorough removal of the culture medium, 300 *μ*L of DMSO was added into each well to dissolve formazan crystals produced by living cells. Then, the DMSO containing formazan crystals was transferred into a 96-well plate (100 *μ*L for each well). After measuring the corresponding OD values in all wells, cell viability of different groups was achieved and analyzed.

### 4.7. In Vitro Vascularization Experiment

HUVECs were bought from the Cell Bank of the Chinese Academy and cultured with Dulbecco's modified Eagle medium (DMEM, Gibco, US) containing 20% fetal bovine serum (FBS) and 1% penicillin-streptomycin. 30 *μ*L/well of growth factor-reduced Matrigel (BD Bioscience) was distributed on the bottom of a 48-well plate, which was placed in an incubator of 37°C for 30 min for sufficient gelation. Suspended HUVECs were then seeded in each well (three wells for each group). Blank culture medium (control group), culture medium including unloaded MXene/PBA hydrogel (experimental group I), and culture medium with adenosine-loaded MXene/PBA hydrogel (experimental group II) were cocultured with the cells. With regard to experimental groups, the MXene-integrated hydrogel patches were irradiated by NIR light for 2 mins. After 6 h of incubation, an optical microscope equipped with a camera was utilized to observe and record the tube formation situation.

### 4.8. Photothermal Experiment

In the photothermal conversion experiments, the microneedle patches with different MXene concentrations were irradiated by a NIR light source (808 nm) with different NIR powers at a distance of 8 cm in liquid or air environment. The corresponding temperature variation was recorded every 15 seconds.

### 4.9. Wound Model Establishment

All animal experiments were approved by the Animal Ethics Committee of Southeast University (No. 20180326003) and were conducted in compliance with the Regulations for the Administration of Affairs Concerning Experimental Animals of China. Firstly, all Sprague-Dawley (SD) rats (200-250 g, male) were anesthetized and randomly divided into three groups that were treated with phosphate buffer solution (control group), PBA/PEGDA microneedle patch without adenosine loading (MN group), PBA/PEGDA microneedle patch encapsulated with adenosine (MN+adenosine group), empty PBA/PEGDA microneedle patch with regular NIR irradiation (MN+NIR group), and adenosine-loaded PBA/PEGDA microneedle patch under regular NIR irradiation (MN+adenosine+NIR group). Afterwards, the round wounds were created by removing the dorsal skin of rats (diameter: around 1 cm). Eight days after the surgery, the rats were sacrificed and the wound tissues were excised for immunofluorescent staining.

### 4.10. Characterization

The morphology of the microneedle patch was observed and recorded by a field emission scanning electron microscope (FESEM, Ultra Plus, Zeiss) after natural drying. The optical images were recorded by an optical microscope (Olympus BX51) which is equipped with a CCD camera (Media Cybernetics Evolution MP5.0). The photothermal effect of the MXene-integrated hydrogel was investigated with near-infrared irradiation (NIR, 808 nm, Xi Long Tech Co., Ltd., China) and studied by the uncooled handheld IR camera (FLIR Systems AB).

### 4.11. Statistics Analysis

Tube formation analysis of HUVECs was conducted by utilizing the plugin (Angiogenesis Analyzer) of the ImageJ software. In addition, the significance of the differences among groups was evaluated by one-way analysis of variance (ANOVA) flowed by the least significant difference (LSD) test of the IBM SPSS Statistics software. For comparison between two groups, the significant differences were calculated by Student's *t*-test (SPSS). When the value of *p* was less than 0.05, the difference was considered as significant.

## Data Availability

All data are contained in the manuscript text and supplementary materials.

## Supplementary Materials

Supplementary Materials Figure S1: component analysis and mechanical test of the MXene-integrated hydrogel. Figure S2: standard curve of adenosine. Figure S3: biocompatibility evaluation of different groups. Figure S4: photothermal property of the MXene-integrated hydrogel in air. Figure S5: results of wound healing process. Figure S6: immunofluorescence staining showing the neovascularization situation of different groups.
