# Supplementary material for: MXene-Integrated Microneedle Patches with Innate Molecule Encapsulation for Wound Healing
Source: Research (Wash D C). 2021 Jun 30;2021:9838490. doi: 10.34133/2021/9838490 (PMC8267825; doi:10.34133/2021/9838490)
Supplement: Supplementary Materials — Figure S1: component analysis and mechanical test of the MXene-integrated hydrogel. Figure S2: standard curve of adenosine. Figure S3: biocompatibility evaluation of different groups. Figure S4: photothermal property of the MXene-integrated hydrogel in air. Figure S5: results of wound healing process. Figure S6: immunofluorescence staining showing the neovascularization situation of different groups. [file 9838490.f1.docx]

**Supplementary**

**MXene-integrated microneedle patches with innate molecule encapsulation for wound healing**

Lingyu Sun^1,2^, Lu Fan^2^, Feika Bian^1^, Guopu Chen^2^, Yuetong Wang^1,2^, Yuanjin Zhao^1,2,^*

^1^ Department of Rheumatology and Immunology, The Affiliated Drum Tower Hospital of Nanjing University Medical School, 210008 Nanjing, China

^2^ State Key Laboratory of Bioelectronics, School of Biological Science and Medical Engineering, Southeast University, Nanjing 210096, China

Email: yjzhao@seu.edu.cn


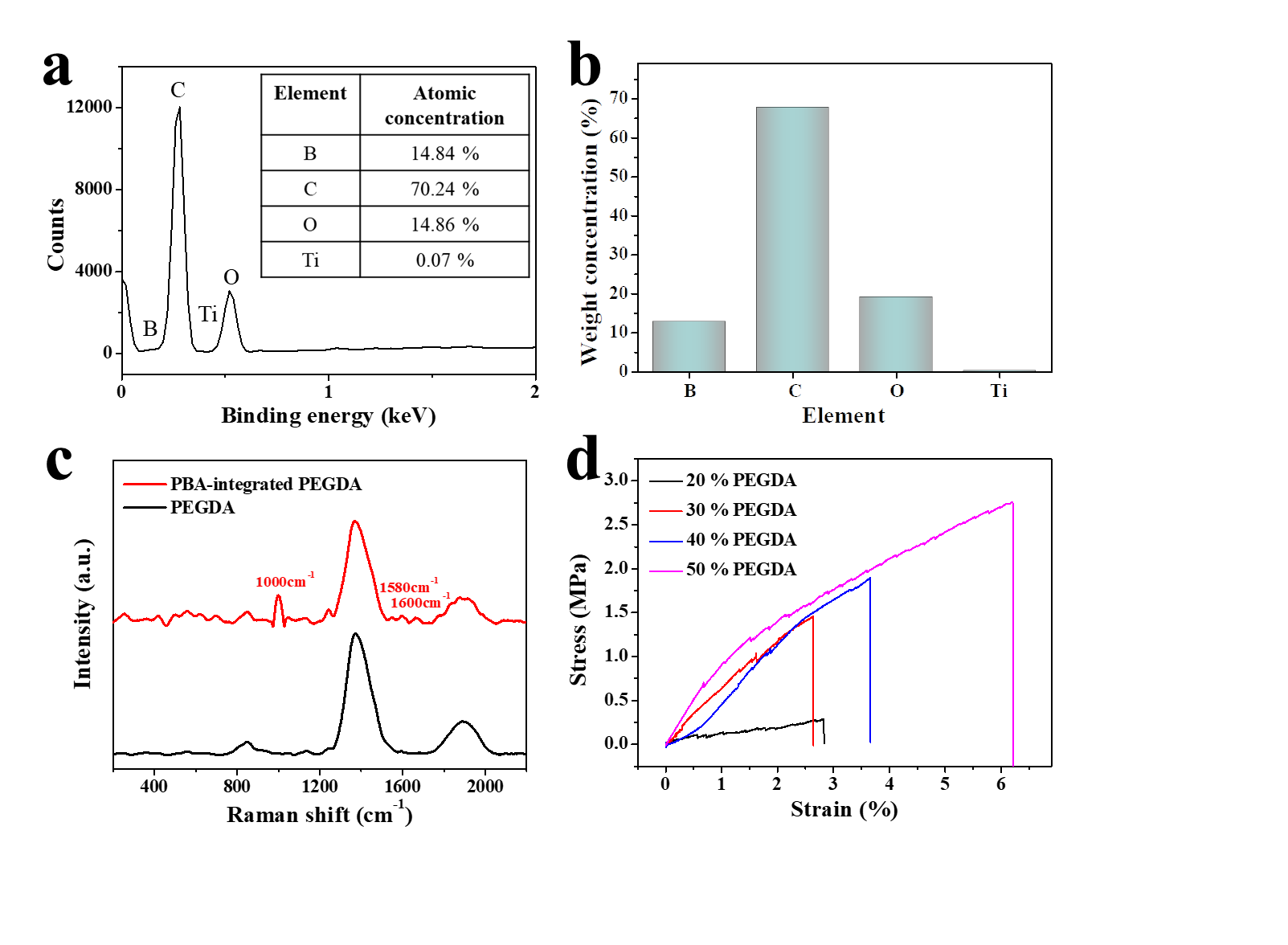


**Figure S1.** **Component analysis and mechanical test of the MXene-integrated hydrogel.** (a) Energy dispersive spectrum of the MXene-integrated PBA hydrogel and its atomic concentrations. (b) Weight concentrations of the composite hydrogel. (c) The Raman spectrums of PBA-integrated PEGDA hydrogel and pure PEGDA hydrogel. (d) Stress-strain curves of the hydrogel with different PEGDA concentrations.


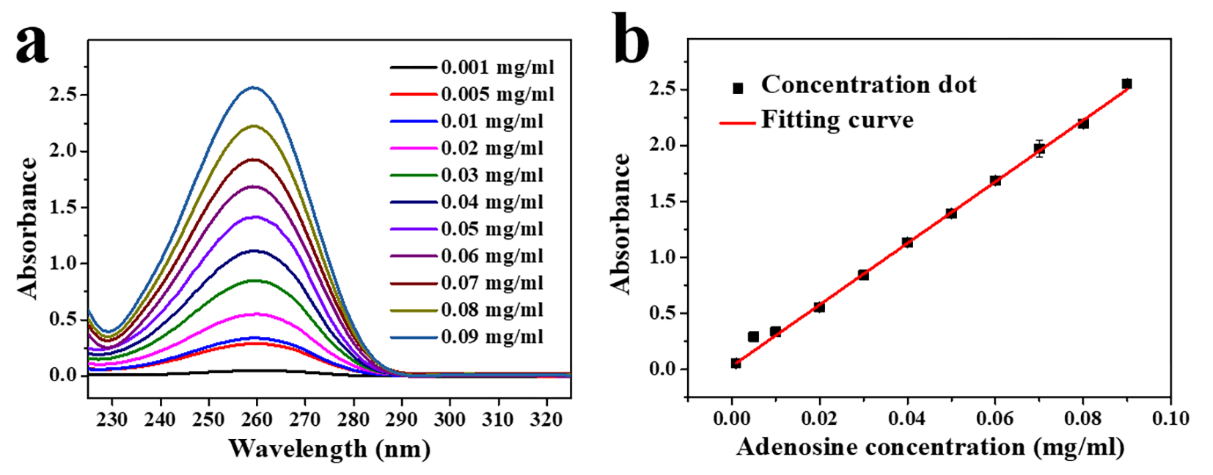


**Figure S2.** **Standard curve of adenosine.** (a) The UV-vis spectra of different concentrations of adenosine (260 nm). (b) Standard curve showing the relationship between absorbance and adenosine concentration.


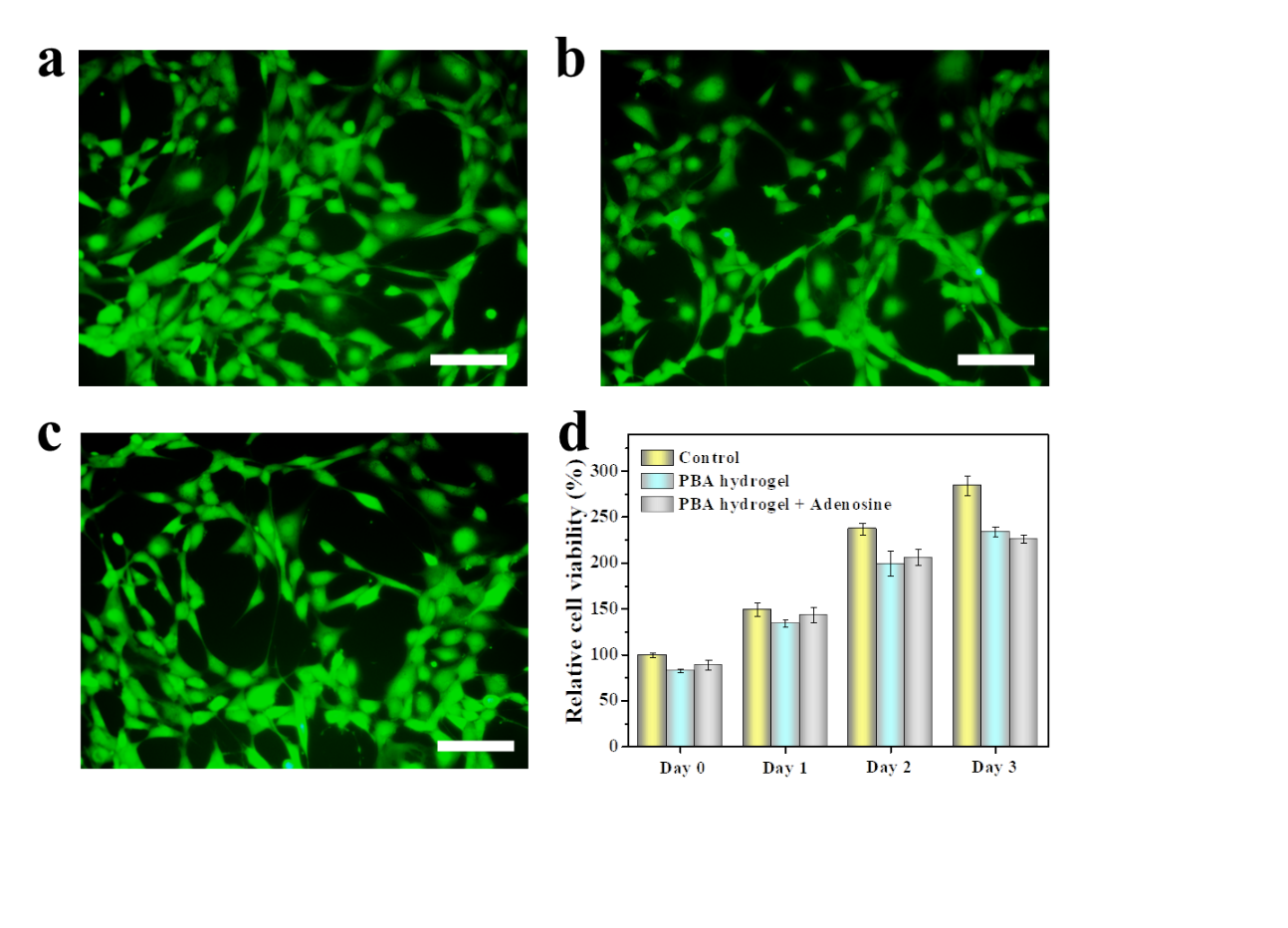


**Figure S3. Biocompatibility evaluation of different groups.** (a-c) Fluorescent images of NIH-3T3 cultured on (a) multi-well plate, (b) multi-well plate with composite hydrogel and (c) multi-well plate with adenosine-loaded composite hydrogel. (d) MTT assay of these three groups. Scale bars are 50 μm.

**
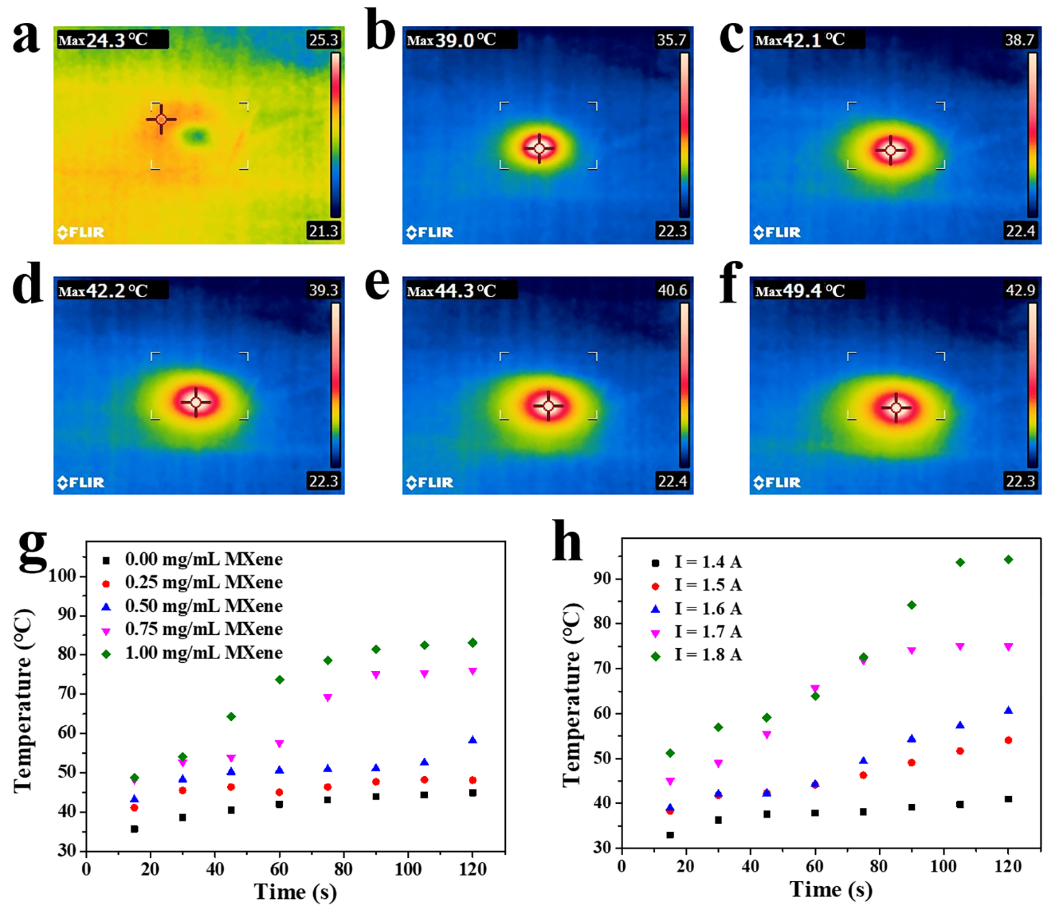
**

**Figure S4.** **Photothermal property of the MXene-integrated hydrogel in air.** (a-f) Temperature increasing process of the MXene-integrated hydrogel under the irradiation of NIR. (g) Temperature-time relationship of the microneedle patch in air under NIR irradiation with different MXene concentrations. (h) Temperature-time curve of the microneedle patch in air under different NIR irradiation intensities.


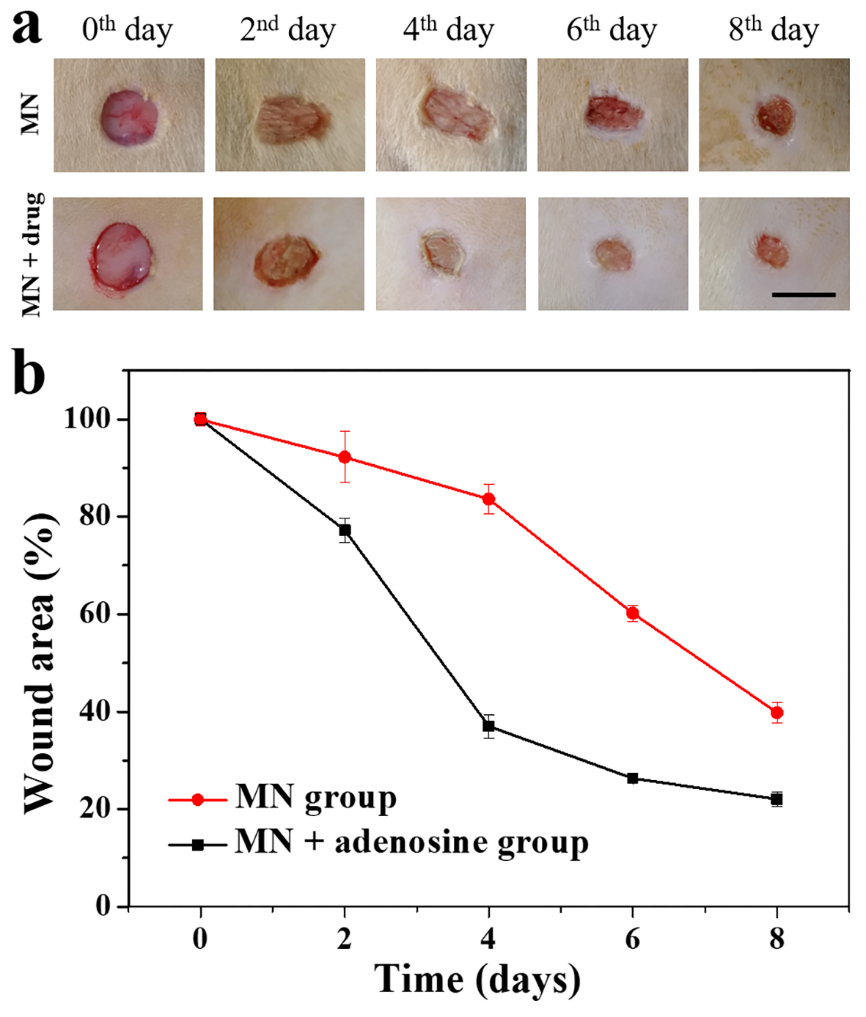


**Figure S5. Results of wound healing process.** (a) Representative images showing the wound healing process in empty patch group and adenosine-loaded patch group (without NIR irradiation). Scale bar is 1 cm. (b) Quantitative analysis of wound area.

**
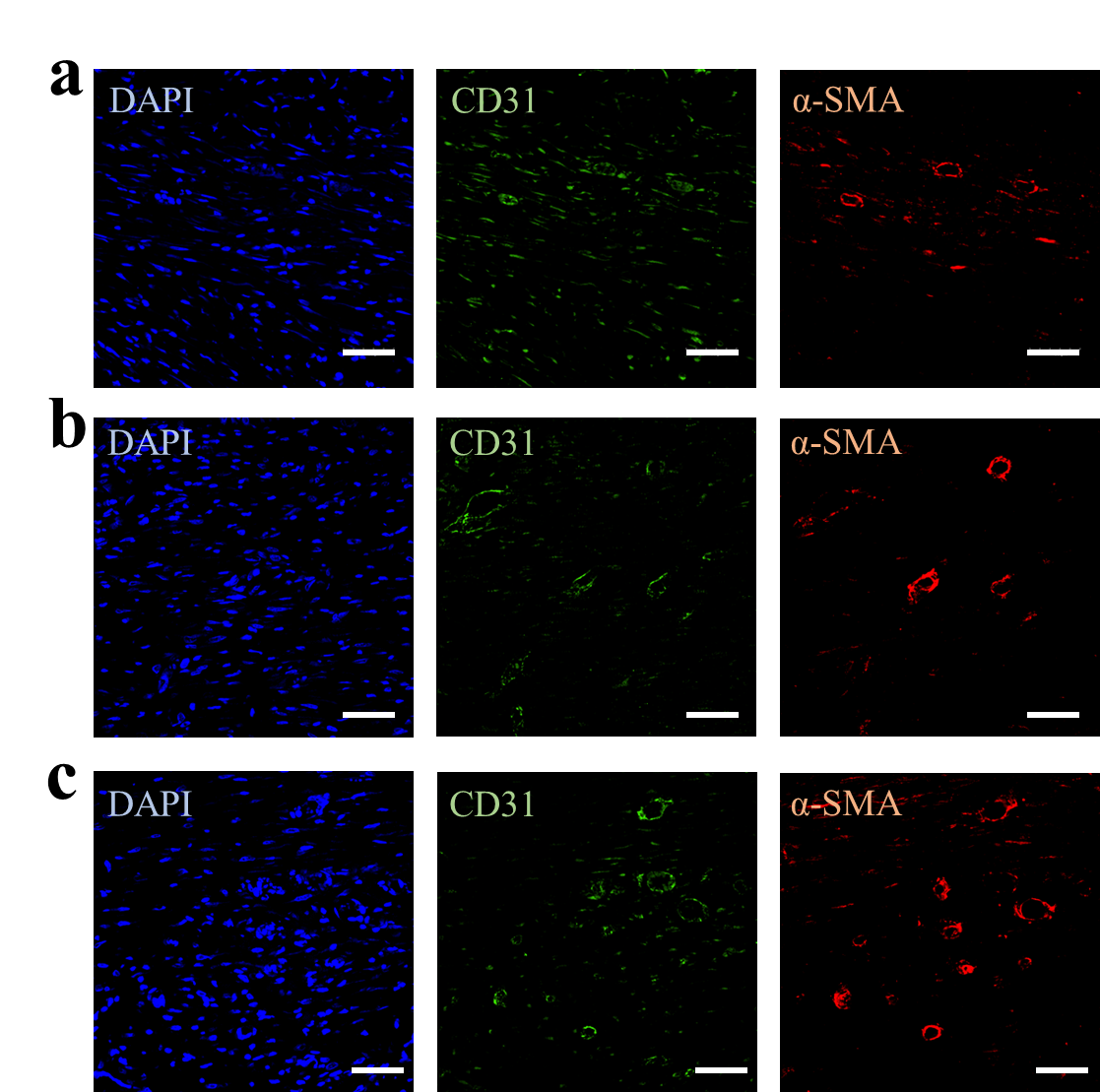
**

Figure S6. Immunofluorescence staining showing the neovascularization situation of different groups. Immunofluorescence staining showing the neovascularization situation of different groups: (a) control group, (b) empty patch group and (c) adenosine-loaded patch group. Scale bars are 50 μm.
